# 4-[(4-Fluoro­benzyl­idene)amino]-3-[1-(4-isobutyl­phen­yl)eth­yl]-1*H*-1,2,4-triazole-5(4*H*)-thione

**DOI:** 10.1107/S160053680903030X

**Published:** 2009-08-08

**Authors:** Hoong-Kun Fun, Wan-Sin Loh, A. C. Vinayaka, B. Kalluraya

**Affiliations:** a X-ray Crystallography Unit, School of Physics, Universiti Sains Malaysia, 11800 USM, Penang, Malaysia; b Department of Studies in Chemistry, Mangalore University, Mangalagangotri, Mangalore 574 199, India

## Abstract

In the title compound, C_21_H_23_FN_4_S, the benzene rings of the isobutyl­phenyl and fluoro­benzene units form dihedral angles of 75.89 (7) and 13.26 (7)°, respectively, with the triazole ring. An intra­molecular C—H⋯S hydrogen-bonding contact generates an *S*(6) ring motif. In the crystal packing, pairs of N—H⋯S hydrogen bonds link neighbouring mol­ecules into inversion dimers, forming *R*_2_^2^(8) ring motifs. The crystal structure is further stabilized by C—H⋯π inter­actions.

## Related literature

For pharmacological activity of triazoles, see: Göknur *et al.* (2005 ▶). For the anti-tumor activity of triazole derivatives, see: Demirbas *et al.* (2002 ▶, 2004 ▶). For the synthesis of related heterocyclic compounds, see: Fun *et al.* (2008 ▶, 2009*a* ▶). For a related structure, see: Fun *et al.* (2009*b* ▶). For hydrogen-bond motifs, see: Bernstein *et al.* (1995 ▶). For the stability of the temperature controller used for the data collection, see: Cosier & Glazer (1986 ▶). 
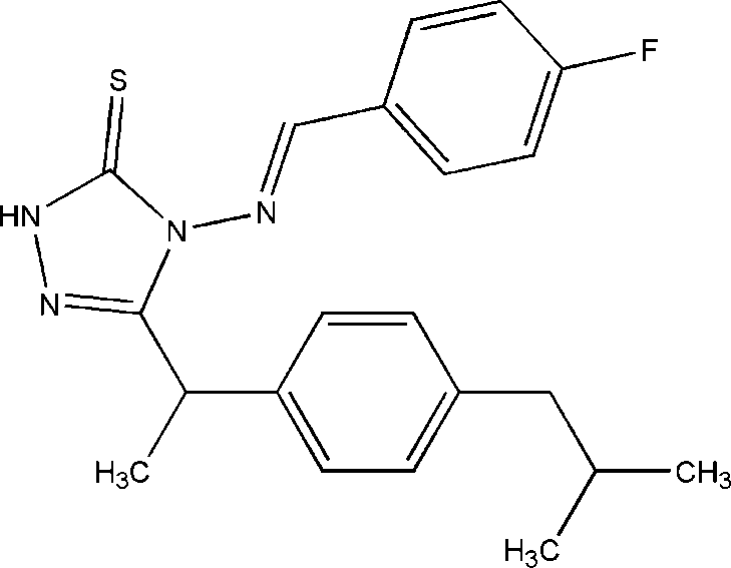


## Experimental

### 

#### Crystal data

C_21_H_23_FN_4_S
*M* *_r_* = 382.49
Triclinic, 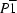

*a* = 5.7883 (1) Å
*b* = 9.9001 (1) Å
*c* = 18.4972 (3) Å
α = 98.132 (1)°
β = 97.087 (1)°
γ = 105.997 (1)°
*V* = 993.90 (3) Å^3^
*Z* = 2
Mo *K*α radiation
μ = 0.19 mm^−1^
*T* = 100 K
0.46 × 0.20 × 0.07 mm

#### Data collection

Bruker SMART APEXII CCD area-detector diffractometer
Absorption correction: multi-scan (**SADABS**; Bruker, 2005 ▶) *T*_min_ = 0.919, *T*_max_ = 0.987
31031 measured reflections
7460 independent reflections
5798 reflections with *I* > 2σ(*I*)
*R*_int_ = 0.037

#### Refinement

*R*[*F* ^2^ > 2σ(*F* ^2^)] = 0.048
*wR*(*F* ^2^) = 0.135
*S* = 1.06
7460 reflections
336 parameters
All H-atom parameters refined
Δρ_max_ = 0.63 e Å^−3^
Δρ_min_ = −0.29 e Å^−3^

### 

Data collection: *APEX2* (Bruker, 2005 ▶); cell refinement: *SAINT* (Bruker, 2005 ▶); data reduction: *SAINT*; program(s) used to solve structure: *SHELXTL* (Sheldrick, 2008 ▶); program(s) used to refine structure: *SHELXTL*; molecular graphics: *SHELXTL*; software used to prepare material for publication: *SHELXTL* and *PLATON* (Spek, 2009 ▶).

## Supplementary Material

Crystal structure: contains datablocks global, I. DOI: 10.1107/S160053680903030X/tk2518sup1.cif

Structure factors: contains datablocks I. DOI: 10.1107/S160053680903030X/tk2518Isup2.hkl

Additional supplementary materials:  crystallographic information; 3D view; checkCIF report

## Figures and Tables

**Table 1:** Hydrogen-bond geometry (Å, °)

*D*—H⋯*A*	*D*—H	H⋯*A*	*D*⋯*A*	*D*—H⋯*A*
N3—H1*N*3⋯S1^i^	0.85 (2)	2.43 (2)	3.2763 (12)	172.3 (18)
C7—H7*A*⋯S1	0.96 (2)	2.50 (2)	3.2415 (13)	133.2 (16)
C4—H4*A*⋯*Cg*1^ii^	1.01 (2)	2.85 (2)	3.6276 (16)	133.8 (17)

Symmetry codes: (i) 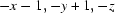; (ii) 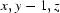. *Cg*1 is the centroid of the C11–C16 ring.

**Fig. 1.**
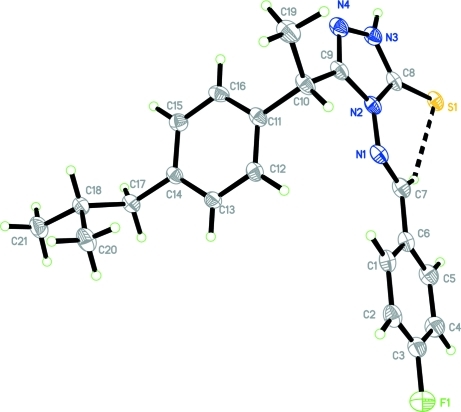
The molecular structure of (I), showing 50% probability displacement ellipsoids and the atom numbering scheme. The intramolecular interaction is shown by a dashed line.

**Fig. 2.**
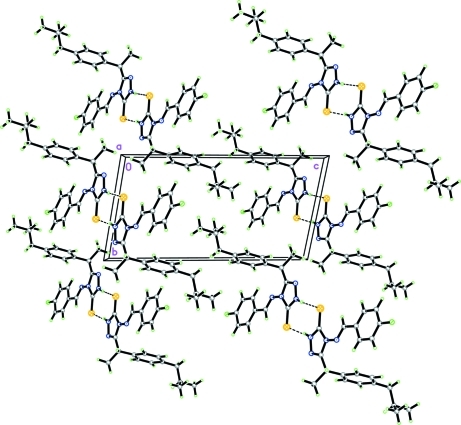
The crystal packing of (I), viewed down the a axis, showing the R22(8) ring motifs. Intermolecular hydrogen bonds are shown by dashed lines.

## supplementary crystallographic information

### Comment

1,2,4-Triazoles and their derivatives represent a rapidly developing field in modern heterocyclic chemistry. Similarly, ibuprofen belongs to the class of Non-Steroidal Anti-Inflammatory Drugs (NSAIDs) with diverse pharmacological activities. The analgesic, anti-asthematic, diuretic, anti-hypertensive and anti-inflammatory properties associated with these drugs have made them important chemotherapeutic agents (Göknur *et al.*, 2005). Our earlier studies involved the synthesis of heterocyclic compounds incorporating ibuprofen and 1,2,4-triazole fragments in the structures (Fun *et al.*, 2008, 2009*a*). Schiff base derivatives of 1,2,4-triazole are known to possess anti-tumor activity (Demirbas *et al.*, 2004). Similarly, some Schiff base derivatives of acetic acid hydrazides containing 1,2,4-triazol-5-one ring have displayed anti-tumoral activity only against breast cancer, while 2-phenyl ethylidenamino and 2-phenyl ethylamino derivatives of 4-amino-1,2,4-triazol-5-ones have been found to be effective towards non-small cell lung cancer, cranial neural crest cancer, and breast cancer (Demirbas *et al.*, 2002). In this connection and in continuation of our interest in the synthesis of chemically and biologically important heterocycles, we now report a substituted 1,2,4-triazole Schiff base carrying the ibuprofen moiety, (I).

In (I), Fig. 1, the triazole ring (C8/C9/N2–N4) is approximately planar with a maximum deviation of 0.009 (1)° at atom N2. The dihedral angles formed by the triazole ring with C1–C6 and C11–C16 benzene rings are 13.26 (7) and 75.89 (7)°, respectively. Bond lengths and angles are comparable to a closely related structure (Fun *et. al.*, 2009*b*). An intramolecular C7—H7A···S1 hydrogen bond generates an *S*(6) ring motif (Bernstein *et al.*, 1995), Fig. 1.

In the crystal packing (Fig. 2), pairs of N3—H1N3···S1 hydrogen bonds link molecules into dimers forming *R*_2_^2^(8) ring motifs; these stack along the *a* axis. The crystal structure is further stabilized by C—H···π interactions (Table 1).

### Experimental

Compound (I) was obtained by refluxing 4-amino-5-[1-(4-isobutylphenyl)ethyl]-4*H*-1,2,4-triazole-3-thiol (0.01 mol) and 4-fluorobenzaldehyde (0.01 mol) in ethanol (50 ml) with 3 drops of concentrated sulfuric acid for 6 h. The solid product obtained was collected by filtration, washed with ethanol and dried. Crystals were obtained from the slow evaporation of an ethanol solution of (I).

### Refinement

All H atoms were located from difference Fourier maps and allowed to refine freely [N—H = 0.85 (2) Å; range of C—H = 0.91 (2) - 1.07 (2) Å].

### Crystal data

**Table:**

C_21_H_23_FN_4_S	*Z* = 2
*M**_r_* = 382.49	*F*(000) = 404
Triclinic, *P*1	*D*_x_ = 1.278 Mg m^−^^3^
Hall symbol: -P 1	Mo *K*α radiation, λ = 0.71073 Å
*a* = 5.7883 (1) Å	Cell parameters from 8265 reflections
*b* = 9.9001 (1) Å	θ = 2.3–32.9°
*c* = 18.4972 (3) Å	µ = 0.19 mm^−^^1^
α = 98.132 (1)°	*T* = 100 K
β = 97.087 (1)°	Plate, colourless
γ = 105.997 (1)°	0.46 × 0.20 × 0.07 mm
*V* = 993.90 (3) Å^3^	

### Data collection

**Table:**

Bruker SMART APEXII CCD area-detector diffractometer	7460 independent reflections
Radiation source: fine-focus sealed tube	5798 reflections with *I* > 2σ(*I*)
graphite	*R*_int_ = 0.037
φ and ω scans	θ_max_ = 33.1°, θ_min_ = 1.1°
Absorption correction: multi-scan (*SADABS*; Bruker, 2005)	*h* = −8→8
*T*_min_ = 0.919, *T*_max_ = 0.987	*k* = −13→15
31031 measured reflections	*l* = −28→28

### Refinement

**Table:**

Refinement on *F*^2^	Primary atom site location: structure-invariant direct methods
Least-squares matrix: full	Secondary atom site location: difference Fourier map
*R*[*F*^2^ > 2σ(*F*^2^)] = 0.048	Hydrogen site location: inferred from neighbouring sites
*wR*(*F*^2^) = 0.135	All H-atom parameters refined
*S* = 1.06	*w* = 1/[σ^2^(*F*_o_^2^) + (0.0657*P*)^2^ + 0.2752*P*] where *P* = (*F*_o_^2^ + 2*F*_c_^2^)/3
7460 reflections	(Δ/σ)_max_ = 0.001
336 parameters	Δρ_max_ = 0.63 e Å^−^^3^
0 restraints	Δρ_min_ = −0.29 e Å^−^^3^

### Special details

**Table:**

Experimental. The crystal was placed in the cold stream of an Oxford Cyrosystems Cobra open-flow nitrogen cryostat (Cosier & Glazer, 1986) operating at 100.0 (1) K.
Geometry. All e.s.d.'s (except the e.s.d. in the dihedral angle between two l.s. planes) are estimated using the full covariance matrix. The cell e.s.d.'s are taken into account individually in the estimation of e.s.d.'s in distances, angles and torsion angles; correlations between e.s.d.'s in cell parameters are only used when they are defined by crystal symmetry. An approximate (isotropic) treatment of cell e.s.d.'s is used for estimating e.s.d.'s involving l.s. planes.
Refinement. Refinement of *F*^2^ against ALL reflections. The weighted *R*-factor *wR* and goodness of fit *S* are based on *F*^2^, conventional *R*-factors *R* are based on *F*, with *F* set to zero for negative *F*^2^. The threshold expression of *F*^2^ > σ(*F*^2^) is used only for calculating *R*-factors(gt) *etc*. and is not relevant to the choice of reflections for refinement. *R*-factors based on *F*^2^ are statistically about twice as large as those based on *F*, and *R*- factors based on ALL data will be even larger.

### Fractional atomic coordinates and isotropic or equivalent isotropic displacement parameters (Å^2^)

**Table:**

	*x*	*y*	*z*	*U*_iso_*/*U*_eq_	
S1	−0.23157 (6)	0.40057 (3)	0.062645 (17)	0.02273 (9)	
F1	1.07698 (17)	0.46076 (11)	0.34727 (5)	0.0384 (2)	
N2	0.09265 (19)	0.67553 (11)	0.11123 (6)	0.0200 (2)	
N3	−0.2432 (2)	0.66569 (12)	0.04603 (6)	0.0240 (2)	
N4	−0.1117 (2)	0.80841 (12)	0.06196 (6)	0.0262 (2)	
C1	0.7243 (2)	0.63478 (14)	0.23907 (7)	0.0230 (2)	
C2	0.9179 (2)	0.61374 (15)	0.28295 (8)	0.0265 (3)	
C3	0.8872 (3)	0.48191 (16)	0.30420 (8)	0.0275 (3)	
C4	0.6725 (3)	0.37142 (15)	0.28390 (8)	0.0291 (3)	
C5	0.4798 (3)	0.39514 (15)	0.24054 (8)	0.0270 (3)	
C6	0.5020 (2)	0.52512 (13)	0.21738 (7)	0.0220 (2)	
C7	0.2896 (2)	0.54128 (14)	0.17189 (7)	0.0239 (2)	
N1	0.29999 (19)	0.65984 (12)	0.15103 (6)	0.0219 (2)	
C8	−0.1269 (2)	0.57973 (13)	0.07367 (6)	0.0207 (2)	
C9	0.0928 (2)	0.81133 (13)	0.10125 (7)	0.0227 (2)	
C10	0.3012 (3)	0.94216 (14)	0.13560 (7)	0.0248 (3)	
C11	0.2842 (2)	0.98403 (13)	0.21717 (7)	0.0210 (2)	
C12	0.4468 (2)	0.96157 (14)	0.27274 (7)	0.0226 (2)	
C13	0.4346 (2)	1.00047 (13)	0.34731 (7)	0.0215 (2)	
C14	0.2607 (2)	1.06384 (13)	0.36891 (7)	0.0198 (2)	
C15	0.0957 (2)	1.08449 (14)	0.31282 (7)	0.0230 (2)	
C16	0.1051 (2)	1.04396 (14)	0.23829 (7)	0.0239 (2)	
C17	0.2509 (2)	1.11216 (14)	0.44918 (7)	0.0232 (2)	
C18	0.3959 (2)	1.26943 (14)	0.48054 (7)	0.0231 (2)	
C19	0.3044 (3)	1.06203 (16)	0.09161 (9)	0.0338 (3)	
C20	0.6693 (3)	1.29519 (17)	0.48410 (9)	0.0302 (3)	
C21	0.3413 (3)	1.31418 (18)	0.55751 (8)	0.0324 (3)	
H1A	0.739 (3)	0.725 (2)	0.2216 (10)	0.031 (4)*	
H2A	1.074 (4)	0.684 (2)	0.2993 (11)	0.042 (5)*	
H4A	0.661 (4)	0.277 (2)	0.3010 (11)	0.042 (5)*	
H5A	0.325 (4)	0.318 (2)	0.2259 (11)	0.038 (5)*	
H7A	0.142 (4)	0.462 (2)	0.1596 (11)	0.037 (5)*	
H10A	0.470 (4)	0.922 (2)	0.1304 (11)	0.035 (5)*	
H12A	0.570 (3)	0.9154 (18)	0.2584 (9)	0.023 (4)*	
H13A	0.545 (3)	0.985 (2)	0.3849 (11)	0.035 (5)*	
H15A	−0.026 (3)	1.1270 (19)	0.3265 (10)	0.030 (4)*	
H16A	−0.007 (4)	1.058 (2)	0.2033 (11)	0.040 (5)*	
H17A	0.314 (3)	1.0534 (19)	0.4791 (10)	0.028 (4)*	
H17B	0.078 (3)	1.0959 (18)	0.4546 (9)	0.024 (4)*	
H18A	0.347 (3)	1.331 (2)	0.4477 (11)	0.035 (5)*	
H19A	0.140 (4)	1.092 (2)	0.0910 (11)	0.044 (5)*	
H19B	0.452 (4)	1.149 (2)	0.1147 (12)	0.047 (6)*	
H19C	0.323 (4)	1.027 (2)	0.0392 (11)	0.040 (5)*	
H20A	0.761 (4)	1.396 (2)	0.5032 (12)	0.047 (6)*	
H20B	0.714 (4)	1.268 (2)	0.4370 (12)	0.041 (5)*	
H20C	0.723 (4)	1.237 (2)	0.5162 (12)	0.043 (5)*	
H21A	0.164 (4)	1.299 (2)	0.5562 (12)	0.048 (6)*	
H21B	0.421 (4)	1.416 (2)	0.5753 (12)	0.042 (5)*	
H21C	0.394 (4)	1.258 (2)	0.5923 (11)	0.036 (5)*	
H1N3	−0.384 (4)	0.641 (2)	0.0196 (11)	0.038 (5)*	

### Atomic displacement parameters (Å^2^)

**Table:**

	*U*^11^	*U*^22^	*U*^33^	*U*^12^	*U*^13^	*U*^23^
S1	0.02380 (15)	0.01846 (14)	0.01925 (15)	−0.00197 (10)	0.00281 (11)	−0.00094 (10)
F1	0.0338 (5)	0.0443 (5)	0.0371 (5)	0.0151 (4)	−0.0013 (4)	0.0066 (4)
N2	0.0209 (5)	0.0188 (5)	0.0150 (4)	−0.0010 (4)	0.0017 (3)	0.0006 (3)
N3	0.0234 (5)	0.0201 (5)	0.0210 (5)	−0.0009 (4)	−0.0016 (4)	−0.0004 (4)
N4	0.0311 (6)	0.0190 (5)	0.0210 (5)	−0.0011 (4)	−0.0011 (4)	0.0012 (4)
C1	0.0241 (6)	0.0198 (5)	0.0216 (6)	0.0017 (4)	0.0074 (4)	−0.0004 (4)
C2	0.0224 (6)	0.0273 (6)	0.0250 (6)	0.0024 (5)	0.0057 (5)	−0.0021 (5)
C3	0.0287 (6)	0.0307 (7)	0.0225 (6)	0.0101 (5)	0.0038 (5)	0.0011 (5)
C4	0.0341 (7)	0.0237 (6)	0.0285 (7)	0.0065 (5)	0.0055 (5)	0.0056 (5)
C5	0.0279 (6)	0.0217 (6)	0.0265 (6)	0.0007 (5)	0.0039 (5)	0.0027 (5)
C6	0.0235 (6)	0.0202 (5)	0.0192 (5)	0.0023 (4)	0.0054 (4)	0.0002 (4)
C7	0.0236 (6)	0.0216 (6)	0.0217 (6)	−0.0002 (4)	0.0041 (5)	0.0017 (4)
N1	0.0207 (5)	0.0236 (5)	0.0171 (5)	0.0009 (4)	0.0028 (4)	0.0015 (4)
C8	0.0211 (5)	0.0209 (5)	0.0143 (5)	−0.0014 (4)	0.0039 (4)	−0.0006 (4)
C9	0.0273 (6)	0.0192 (5)	0.0159 (5)	−0.0012 (4)	0.0024 (4)	0.0020 (4)
C10	0.0281 (6)	0.0208 (6)	0.0174 (5)	−0.0035 (5)	0.0018 (5)	0.0013 (4)
C11	0.0227 (5)	0.0161 (5)	0.0178 (5)	−0.0029 (4)	0.0007 (4)	0.0018 (4)
C12	0.0233 (6)	0.0199 (5)	0.0211 (6)	0.0033 (4)	0.0020 (4)	0.0005 (4)
C13	0.0232 (6)	0.0198 (5)	0.0189 (5)	0.0049 (4)	−0.0007 (4)	0.0024 (4)
C14	0.0198 (5)	0.0175 (5)	0.0182 (5)	0.0004 (4)	0.0015 (4)	0.0020 (4)
C15	0.0183 (5)	0.0237 (6)	0.0246 (6)	0.0038 (4)	0.0007 (4)	0.0036 (5)
C16	0.0209 (6)	0.0245 (6)	0.0215 (6)	0.0016 (4)	−0.0035 (4)	0.0052 (5)
C17	0.0230 (6)	0.0245 (6)	0.0199 (6)	0.0039 (5)	0.0038 (4)	0.0029 (5)
C18	0.0235 (6)	0.0239 (6)	0.0198 (6)	0.0073 (4)	0.0004 (4)	0.0000 (4)
C19	0.0446 (9)	0.0252 (7)	0.0250 (7)	−0.0006 (6)	0.0034 (6)	0.0071 (5)
C20	0.0236 (6)	0.0323 (7)	0.0277 (7)	0.0019 (5)	0.0037 (5)	−0.0040 (6)
C21	0.0293 (7)	0.0391 (8)	0.0261 (7)	0.0126 (6)	0.0033 (5)	−0.0062 (6)

### Geometric parameters (Å, °)

**Table:**

S1—C8	1.6821 (13)	C11—C12	1.3904 (18)
F1—C3	1.3577 (16)	C11—C16	1.3976 (19)
N2—C9	1.3824 (16)	C12—C13	1.3945 (18)
N2—N1	1.3870 (15)	C12—H12A	0.989 (16)
N2—C8	1.3888 (15)	C13—C14	1.3935 (17)
N3—C8	1.3389 (17)	C13—H13A	0.941 (19)
N3—N4	1.3772 (15)	C14—C15	1.3980 (17)
N3—H1N3	0.85 (2)	C14—C17	1.5071 (17)
N4—C9	1.3011 (17)	C15—C16	1.3923 (19)
C1—C2	1.382 (2)	C15—H15A	0.958 (18)
C1—C6	1.4055 (18)	C16—H16A	0.91 (2)
C1—H1A	0.977 (18)	C17—C18	1.5392 (19)
C2—C3	1.387 (2)	C17—H17A	0.968 (18)
C2—H2A	0.96 (2)	C17—H17B	0.988 (17)
C3—C4	1.379 (2)	C18—C20	1.524 (2)
C4—C5	1.385 (2)	C18—C21	1.5272 (19)
C4—H4A	1.01 (2)	C18—H18A	0.989 (19)
C5—C6	1.3908 (19)	C19—H19A	1.07 (2)
C5—H5A	0.98 (2)	C19—H19B	1.03 (2)
C6—C7	1.4618 (19)	C19—H19C	1.01 (2)
C7—N1	1.2747 (17)	C20—H20A	0.98 (2)
C7—H7A	0.97 (2)	C20—H20B	0.96 (2)
C9—C10	1.5013 (17)	C20—H20C	0.97 (2)
C10—C19	1.528 (2)	C21—H21A	0.99 (2)
C10—C11	1.5299 (18)	C21—H21B	0.97 (2)
C10—H10A	1.059 (19)	C21—H21C	0.98 (2)
			
C9—N2—N1	118.18 (10)	C11—C12—H12A	118.7 (10)
C9—N2—C8	108.16 (11)	C13—C12—H12A	120.3 (10)
N1—N2—C8	133.59 (11)	C14—C13—C12	121.45 (11)
C8—N3—N4	114.43 (11)	C14—C13—H13A	117.7 (12)
C8—N3—H1N3	127.2 (13)	C12—C13—H13A	120.9 (12)
N4—N3—H1N3	118.4 (13)	C13—C14—C15	117.37 (11)
C9—N4—N3	103.92 (11)	C13—C14—C17	122.28 (11)
C2—C1—C6	120.41 (13)	C15—C14—C17	120.33 (11)
C2—C1—H1A	121.3 (11)	C16—C15—C14	121.37 (12)
C6—C1—H1A	118.2 (11)	C16—C15—H15A	120.0 (11)
C1—C2—C3	118.32 (13)	C14—C15—H15A	118.7 (11)
C1—C2—H2A	124.2 (12)	C15—C16—C11	120.84 (12)
C3—C2—H2A	117.5 (12)	C15—C16—H16A	119.0 (13)
F1—C3—C4	118.41 (13)	C11—C16—H16A	120.2 (13)
F1—C3—C2	118.51 (13)	C14—C17—C18	114.35 (11)
C4—C3—C2	123.08 (13)	C14—C17—H17A	109.5 (10)
C3—C4—C5	117.71 (13)	C18—C17—H17A	107.9 (11)
C3—C4—H4A	119.6 (12)	C14—C17—H17B	108.3 (10)
C5—C4—H4A	122.6 (12)	C18—C17—H17B	109.7 (10)
C4—C5—C6	121.40 (13)	H17A—C17—H17B	106.9 (14)
C4—C5—H5A	119.0 (11)	C20—C18—C21	109.98 (11)
C6—C5—H5A	119.6 (11)	C20—C18—C17	111.77 (11)
C5—C6—C1	119.08 (13)	C21—C18—C17	110.13 (12)
C5—C6—C7	117.86 (12)	C20—C18—H18A	107.0 (11)
C1—C6—C7	123.06 (12)	C21—C18—H18A	108.4 (11)
N1—C7—C6	119.92 (12)	C17—C18—H18A	109.4 (12)
N1—C7—H7A	121.5 (11)	C10—C19—H19A	111.1 (11)
C6—C7—H7A	118.5 (11)	C10—C19—H19B	109.4 (12)
C7—N1—N2	119.06 (11)	H19A—C19—H19B	109.8 (16)
N3—C8—N2	102.50 (10)	C10—C19—H19C	107.9 (11)
N3—C8—S1	126.62 (10)	H19A—C19—H19C	109.8 (15)
N2—C8—S1	130.88 (10)	H19B—C19—H19C	108.7 (17)
N4—C9—N2	110.96 (11)	C18—C20—H20A	111.5 (12)
N4—C9—C10	126.38 (12)	C18—C20—H20B	113.4 (12)
N2—C9—C10	122.59 (12)	H20A—C20—H20B	108.1 (18)
C9—C10—C19	110.48 (11)	C18—C20—H20C	109.1 (13)
C9—C10—C11	108.88 (10)	H20A—C20—H20C	109.1 (17)
C19—C10—C11	113.34 (11)	H20B—C20—H20C	105.5 (17)
C9—C10—H10A	110.3 (11)	C18—C21—H21A	111.1 (13)
C19—C10—H10A	103.0 (11)	C18—C21—H21B	110.1 (13)
C11—C10—H10A	110.7 (11)	H21A—C21—H21B	106.5 (17)
C12—C11—C16	117.98 (12)	C18—C21—H21C	110.7 (11)
C12—C11—C10	120.42 (12)	H21A—C21—H21C	107.5 (17)
C16—C11—C10	121.60 (11)	H21B—C21—H21C	110.9 (17)
C11—C12—C13	120.95 (12)		
			
C8—N3—N4—C9	−0.39 (15)	C8—N2—C9—N4	1.56 (14)
C6—C1—C2—C3	0.56 (19)	N1—N2—C9—C10	−3.85 (17)
C1—C2—C3—F1	179.96 (12)	C8—N2—C9—C10	178.80 (11)
C1—C2—C3—C4	−0.3 (2)	N4—C9—C10—C19	−26.92 (19)
F1—C3—C4—C5	179.39 (12)	N2—C9—C10—C19	156.28 (13)
C2—C3—C4—C5	−0.3 (2)	N4—C9—C10—C11	98.16 (15)
C3—C4—C5—C6	0.8 (2)	N2—C9—C10—C11	−78.64 (15)
C4—C5—C6—C1	−0.5 (2)	C9—C10—C11—C12	107.13 (14)
C4—C5—C6—C7	−179.87 (13)	C19—C10—C11—C12	−129.48 (14)
C2—C1—C6—C5	−0.16 (19)	C9—C10—C11—C16	−72.11 (15)
C2—C1—C6—C7	179.16 (12)	C19—C10—C11—C16	51.28 (17)
C5—C6—C7—N1	178.23 (12)	C16—C11—C12—C13	−1.31 (18)
C1—C6—C7—N1	−1.1 (2)	C10—C11—C12—C13	179.42 (11)
C6—C7—N1—N2	−176.62 (11)	C11—C12—C13—C14	−0.41 (19)
C9—N2—N1—C7	167.93 (12)	C12—C13—C14—C15	1.20 (18)
C8—N2—N1—C7	−15.5 (2)	C12—C13—C14—C17	−177.44 (12)
N4—N3—C8—N2	1.29 (14)	C13—C14—C15—C16	−0.28 (18)
N4—N3—C8—S1	−178.22 (9)	C17—C14—C15—C16	178.39 (12)
C9—N2—C8—N3	−1.65 (13)	C14—C15—C16—C11	−1.5 (2)
N1—N2—C8—N3	−178.43 (12)	C12—C11—C16—C15	2.23 (18)
C9—N2—C8—S1	177.83 (10)	C10—C11—C16—C15	−178.51 (11)
N1—N2—C8—S1	1.1 (2)	C13—C14—C17—C18	92.42 (15)
N3—N4—C9—N2	−0.73 (14)	C15—C14—C17—C18	−86.18 (14)
N3—N4—C9—C10	−177.84 (12)	C14—C17—C18—C20	−66.53 (15)
N1—N2—C9—N4	178.91 (11)	C14—C17—C18—C21	170.91 (11)

### Hydrogen-bond geometry (Å, °)

**Table:**

*D*—H···*A*	*D*—H	H···*A*	*D*···*A*	*D*—H···*A*
N3—H1N3···S1^i^	0.85 (2)	2.43 (2)	3.2763 (12)	172.3 (18)
C7—H7A···S1	0.96 (2)	2.50 (2)	3.2415 (13)	133.2 (16)
C4—H4A···Cg1^ii^	1.01 (2)	2.85 (2)	3.6276 (16)	133.8 (17)

Symmetry codes: (i) −*x*−1, −*y*+1, −*z*; (ii) *x*, *y*−1, *z*.
